# A Pilot Randomized Control Trial Evaluating the Feasibility of a 12-Week Mediterranean Diet Intervention Without Caloric Restriction in Women with Polycystic Ovary Syndrome

**DOI:** 10.3390/jcm14165842

**Published:** 2025-08-18

**Authors:** Nicole Scannell, Evangeline Mantzioris, Stephanie Cowan, Lisa Moran, Anthony Villani

**Affiliations:** 1 School of Health, University of the Sunshine Coast, Sippy Downs, QLD 4556, Australia; nicole.scannell@research.usc.edu.au (N.S.); avillani@usc.edu.au (A.V.); 2 UniSA: Clinical & Health Sciences, Alliance for Research in Exercise, Nutrition, and Activity (ARENA), University of South Australia, Adelaide, SA 5000, Australia; evangeline.mantzioris@unisa.edu.au; 3 Monash Centre for Health Research and Implementation (MCHRI), School of Public Health and Preventive Medicine, Monash University, Melbourne, VIC 3800, Australia; stephanie.cowan@monash.edu

**Keywords:** feasibility, dietary adherence, PCOS, mediterranean diet, pilot study

## Abstract

**Background/Objectives**: Women with Polycystic Ovary Syndrome (PCOS) often report difficulties adhering to dietary interventions due to a combination of physiological and psychological barriers. Therefore, this study explores the feasibility of a Mediterranean diet (MedDiet) intervention as an effective and acceptable dietary approach for managing PCOS. **Methods**: Women with PCOS and a BMI ≥ 25 kg/m^2^, aged 18–45 years were randomized to an ad libitum MedDiet or Healthy Eating (HE) diet (control). The 12-week intervention incorporated fortnightly, personalized dietary consultations and tailored resources. Primary outcomes were measures of feasibility, including recruitment metrics, data collection methods, and intervention adherence. Acceptability was examined using semi-structured interviews and surveys for those randomized to the MedDiet. **Results**: Study promotion resulted in *n =* 380 interested individuals; a total of *n* = 26 were randomized to either a MedDiet (*n* = 12) or HE (*n* = 14) group. Data collection was mostly appropriate as demonstrated by the collection of 100% of anthropometric and biochemical data; however, only 69% of the 4-day food records were returned. Participants reported the intervention was acceptable, and adherence was enhanced through individualized counselling and the provision of practical resources. At week 12, adherence was significantly greater in the MedDiet group compared to HE (8.1 ± 2.4 vs. 4.6 ± 1.0; *p* = 0.002). Adherence significantly improved from baseline to week 12 in both groups (MedDiet: 3.67 ± 1.32; 8.11 ± 2.37; *p* ≤ 0.001; HE: 3.57 ± 1.27; 4.57 ± 0.98; *p* = 0.02). **Conclusions**: We showed that a 12-week pilot MedDiet intervention is feasible and acceptable for women with PCOS and a BMI ≥ 25 kg/m^2^. Future investigation warrants a larger, adequately powered study which addresses challenges to recruitment, attrition and collection of dietary intake data.

## 1. Introduction

Polycystic Ovary Syndrome (PCOS) is a common endocrine condition, affecting approximately 8–13% of women of reproductive age [1]. It is a complex condition characterised by reproductive, metabolic and psychological features [2,3,4]. These features include oligo-/anovulation, contributing to infertility and increased risks of endometrial cancer, and metabolic concerns including an increased risk for type 2 diabetes and cardiovascular disease (CVD) [5,6,7,8]. Furthermore, women with PCOS experience higher rates of depression, anxiety [9,10] and disordered eating behaviours compared to their healthy counterparts [11,12].

Insulin resistance in PCOS is a key aetiological feature in PCOS. This is twofold: firstly, intrinsic IR underpins the condition, negatively affecting hormonal and clinical features. Secondly, extrinsic IR can be triggered by excess weight, further exacerbating PCOS features and symptoms [13,14]. This is further compounded by women with PCOS having an increased prevalence of overweight, central adiposity, and weight gain over time [15,16].

The International PCOS guidelines recommend lifestyle management (diet, physical activity and behavioural strategies) as well as weight management (weight gain prevention, modest weight loss, weight maintenance) for all women [17]. Following a healthy diet is considered one of the first line management strategies and is widely recognised for mitigating the long-term health risks that accompany PCOS [1,4]. As there is no evidence to support one superior dietary approach for PCOS management, current international PCOS guidelines recommend adherence to a dietary pattern that is consistent with general population-based dietary guidelines [18]. However, many women with PCOS have expressed the desire for more tailored dietary recommendations that address the unique hormonal challenges associated with the condition [19,20]. At present, the research literature on dietary interventions for PCOS management has predominantly focused on caloric restriction to promote weight loss [21,22]. While weight management may be an appropriate strategy for some individuals, for others, it may not align with their health history [11] or personal health priorities [23]. For example, a higher prevalence of cardiometabolic risk factors has been observed in individuals with PCOS, independent of BMI and adiposity [24,25]. Moreover, the sustainability of weight loss interventions has been questioned [26], particularly given high attrition rates [27], suggesting such interventions may not be practical in real-world settings. In a review examining dietary and lifestyle interventions aimed at reducing cardiometabolic risk factors in women with PCOS, Herbert and Woolf [26] reported that studies which did modify dietary patterns, without caloric restriction, still demonstrated improvements in cardiometabolic risk factors, suggesting less restrictive dietary approaches may still be effective at reducing cardiometabolic disease risk. Additionally, most dietary studies recruiting women with PCOS tend to rely on BMI (e.g., to identify overweight and obesity) which may limit our understanding of best practice lifestyle approaches for women across all body sizes [26]. Of note, individuals who are classified as either ‘overweight’ or ‘obese’ in accordance with BMI categories may still be metabolically healthy; in contrast, individuals classified into a ‘healthy’ BMI category could still be metabolically unhealthy [28]. Given that there is likely no ‘one-size-fits-all’ approach for PCOS management, removing weight stigma and exploring alternative dietary approaches that extend beyond weight loss as the primary aim, particularly amongst women living in larger bodies is warranted.

In populations with similar health vulnerabilities to women with PCOS, there is evidence showing that greater adherence to a Mediterranean-style diet is inversely associated with central adiposity and weight loss (independent of caloric restriction) in epidemiological and dietary intervention studies, respectively [29,30]. Furthermore, adherence to a Mediterranean diet (MedDiet) has been associated with improved insulin sensitivity, glycaemic control, and a reduction in depressive symptoms, particularly among those with metabolic disorders [31]. Furthermore, there is emerging evidence suggesting that a MedDiet may offer protective benefits in PCOS management [32]. Observational studies have reported that greater MedDiet adherence is inversely associated with hyperandrogenism and IR [33]. Moreover, in a case–control study, Cutillas-Tolin [34] reported that adherence to a MedDiet was protective against hyperandrogenism and oligo-/anovulation. Nevertheless, evidence from clinical trials is scant. One of the few randomised controlled trials (RCTs) to investigate the benefits of a MedDiet intervention on PCOS features demonstrated that in women with overweight or obesity, a low-energy, moderate carbohydrate, Mediterranean-style diet was more effective than a calorie-restricted, low-fat diet in reducing body weight and waist circumference as well as cardiometabolic and hormonal parameters, including fasting blood glucose, insulin sensitivity, blood lipid levels, total testosterone, and luteinizing hormone concentrations [35]. These findings suggest a MedDiet has the potential to offer a sustainable lifestyle solution for PCOS management.

Given that women with PCOS frequently report difficulties with adhering to lifestyle management, including dietary change [19], it would be beneficial for future dietary interventions to evaluate not only the efficacy but also the acceptability and feasibility of dietary interventions within this population. We have shown that despite some barriers, a Mediterranean-style diet may be a viable and sustainable dietary approach for women with PCOS, particularly when it is supported with ongoing dietary counselling and behavioural strategies that address practical barriers and enhance motivational factors [36]. Preliminary studies are often used to explore the feasibility of dietary interventions before advancing to larger trials [37,38] as findings from such investigations can enhance the effectiveness of future RCTs by identifying potential challenges [39]. As such, the aim of this study was to evaluate the feasibility of a pilot ad libitum MedDiet intervention for improving metabolic, hormonal and anthropometric outcomes among women with PCOS and a BMI ≥ 25 kg/m^2^. This study seeks to inform the design of a larger, adequately powered clinical trial by addressing the following research objectives:

1. Assess participant recruitment to an ad libitum MedDiet intervention among women with PCOS and a BMI ≥ 25 kg/m^2^, including recruitment strategies and eligibility.
2. Evaluate the appropriateness and reliability of data collection methods for capturing key outcomes related metabolic, hormonal, anthropometric, and dietary adherence.
3. Examine the acceptability and practicality of the MedDiet intervention, including participants’ satisfaction, and perceived barriers and facilitators to dietary adherence.
4. Conduct a preliminary assessment of intervention effects on metabolic, hormonal and anthropometric measures and safety of the MedDiet intervention.

## 2. Materials and Methods

### 2.1. Study Reporting

The reporting of this pilot feasibility study followed the CONSORT guidelines for pilot and feasibility trials [40] and incorporated recommendations from Pfledderer et al. [41] for behavioural intervention pilot and feasibility studies. The study was registered with the Australian and New Zealand Clinical Trials Registry (ACTRN12621000994886) and conducted in accordance with the Declaration of Helsinki. Ethical approval was obtained from the Human Research Ethics Committees at the University of the Sunshine Coast (A211524), the University of South Australia (203950), and Monash University (29090). This paper presents results from a feasibility perspective only. A comprehensive study protocol has been published elsewhere [42].

### 2.2. Study and Intervention Design

This was a 12-week pilot randomized controlled trial (RCT) evaluating the effects of a MedDiet intervention, without caloric restriction, on hormonal, metabolic, and anthropometric outcomes in women (18–45 years) with PCOS and a BMI of ≥25 kg/m^2^. This multi-centre RCT took place between July 2021 and May 2024 at the University of the Sunshine Coast, Monash University and the University of South Australia.

#### Stakeholder Engagement

Study resources, intervention procedures, and surveys were developed by the research team in consultation with a stakeholder advisory committee comprising an obstetrician-gynaecologist, an endocrinologist, an Accredited Practising Dietitian, and a consumer representative. Members were selected for their expertise in PCOS research, clinical care, and lived experience. Meetings were conducted online, during protocol development and study recruitment to provide strategic input and to review, discuss, and pilot questionnaires to provide face validity [43].

### 2.3. Participants and Recruitment

#### 2.3.1. Inclusion Criteria

Participants were eligible for inclusion if they had a written confirmation of a PCOS diagnosis from a medical doctor, a BMI of ≥25 kg/m^2^, were aged between 18 and 45 years, and were not currently pregnant. Exclusion criteria included the use of insulin-sensitising medications or hormonal contraceptives within three months before the trial commenced, as well as medical conditions such as Cushing’s syndrome, type 1 or type 2 diabetes, thyroid disorders, active cancer, or adrenal tumours. Additionally, individuals with high adherence to a MedDiet, as defined by a Mediterranean diet adherence screener (MEDAS) score [44] ≥ 10, were excluded.

Participants were recruited through a rolling recruitment process. Initially, recruitment was planned between June 2021 and June 2022; however, this period was extended to January 2024 to allow for additional enrolment. Recruitment strategies included advertisements in PCOS-specific social media support groups, paid social media advertising, community posters, collaboration with local medical and allied health clinics, and paid promotional materials distributed at local health and fitness expos.

Interested individuals could inquire about the study via phone, email, or direct social media messaging. They were then provided with a standardised study outline and research project information sheet, detailing the inclusion and exclusion criteria, study duration, and appointment availability, either in written form or verbally. Those expressing continued interest underwent formal eligibility screening using a standardised form assessing against the aforementioned inclusion/exclusion criteria. Eligible participants were then invited to schedule a baseline appointment. For those who initially expressed interest but did not complete the formal screening process, the investigators made a minimum of three follow-up contact attempts to seek interest and facilitate participation.

#### 2.3.2. Sample Size

Sample size calculations were based on expected changes in HOMA-IR as reported by Ryan et al. [45]. To achieve 80% power at a significance level of *p* < 0.05 (two-sided), a total of *n* = 32 participants were required to detect a mean difference of 1.7 ± 0.5. Considering an estimated attrition rate of 30%, commonly reported in weight-loss interventions, the target sample size was set at *n* = 42 participants (*n* = 21 per group).

#### 2.3.3. Randomization

Participants were randomised to receive either a MedDiet intervention or a Healthy Eating (HE) control, which was consistent with standard population-based dietary guidelines, and current dietary recommendations for PCOS as per the current iteration of the international evidence-based guidelines [18]. Randomisation was stratified according to BMI (25–29.9 kg/m^2^ vs. ≥30 kg/m^2^) using a computer-generated randomisation schedule, managed by an independent researcher at the University of the Sunshine Coast. Allocations were assigned by a single researcher and Accredited Practising Dietitian (NS) at the baseline appointment.

#### 2.3.4. Setting

Participants attended clinic spaces at the University of the Sunshine Coast, the University of South Australia, or Monash University for the baseline and week 12 assessments. These spaces were designed to reflect potential clinic settings for a larger-scale study and were equipped with appropriate anthropometric measurement tools and private, quiet rooms for confidential consultations. Subsequent fortnightly consultations (weeks 2, 4, 6, 8 and 10) were offered at the Sunshine Coast site or online. For participants opting for online consultations, sessions were conducted via Zoom, allowing them to attend from a location of their choice. All consultations were conducted by the same Accredited Practising Dietitian. Biochemical testing was carried out by external laboratories, with participants required to visit these facilities either the day before or on the same day as their initial baseline appointment, and again at study completion (week 12). Laboratories were available in multiple locations, including sites near the in-person consultation centres, to facilitate accessibility.

### 2.4. Dietary Intervention

The intervention was delivered by the same Accredited Practising Dietitian (NS) over 12 weeks and standardised case report forms were used throughout each consultation to maximise standardisation of the dietary intervention. Table 1 presents the intervention overview. Both groups received the same number and type of consultation schedule, individual support, and resources.

#### 2.4.1. Theory

The intervention framework and analysis were informed by the Capability, Opportunity, Motivation—Behaviour (COM-B) model and incorporated behaviour change techniques based on an established taxonomy [46] to systematically address behavioural factors influencing dietary change (Table 1).

**Table 1:** Study overview according to the template of TiDIER [47] and aligned with behaviour change techniques [46].

Name	Ad Libitum Mediterranean Diet or Healthy Eating Intervention	Behaviour Change Techniques
What materials	Health information, meal suggestions and recipes, fridge magnet, shopping list, infographic, food checklists. Weekly one-way digital messages to provide health prompts. Content of messages addressed motivational, educational, practical and social aspects.	7.1 Prompts/cues 12.5 Adding objects to the environment 5.1 Information about health consequences 4.1 Instructions on how to perform a behaviour 2.3 Self-monitoring of behaviour 13.2 Framing/reframing 4.2 Information about antecedents 11.2 Reduce negative emotions 1.4 Action planning 3.3 Social support (emotional)—MI 3.1 Social support (unspecified)
What procedures	One-on-one fortnightly dietary consultations Structured education presented using PowerPoint slides	1.1 Goal setting 1.4 Action planning 1.5 Review behaviour goal 4.1 Instructions on how to perform a behaviour 1.2 Problem solving 5.1 Information about health consequences
Who provided	Accredited Practising Dietitian (NS)	9.1 Credible source
How	Face to face and online via Zoom according to participant location and preference	
Where	The Accredited Practising Dietitian provided consultations from the University clinic room	
When and how much	Baseline (90 min) Week 12 (60–90 min) Weeks 2, 4, 6, 8 (10–30 min)	
Tailoring	Dietary counselling, meal suggestions, recipes and adaptations were provided based on the needs of the participant. For example, meal ideas could be modified based on taste preference, dietary requirements, cooking skills and time constraints.	4.1 Instructions on how to perform a behaviour 1.2 Problem solving
Modifications	Due to the geographic distribution of the additional study sites, the baseline appointment for these participants was conducted via Zoom, and study resource materials were mailed to participants.	
How well	The same Accredited Practising Dietitian delivered all dietary intervention information and consultations. Standardised case notes were completed at each consultation, adhering to the predetermined fortnightly structure to ensure consistency in data collection and intervention delivery.	

#### 2.4.2. Dietary Protocol

Participants were randomly allocated to either an ad libitum MedDiet or a Healthy Eating (HE) control. Participants allocated to the HE control were encouraged to adhere to a dietary pattern consistent with the Australian Dietary Guidelines and the Australian Guide to Healthy Eating. This dietary pattern emphasizes a greater intake of wholegrain breads and cereals, an abundance of vegetables and fruit, low-fat dairy products, lean protein sources such as poultry, fish, and lean meats, and smaller amounts of unsaturated fats.

The MedDiet intervention was based on traditional MedDiet principles, whilst adapted to accommodate the dietary habits and cultural preferences of the Australian population. This approach prioritizes a high intake and diverse selection of plant-based foods, including fruits, vegetables, wholegrain cereals, legumes, nuts and the exclusive use of extra virgin olive oil (EVOO). Consistent with this dietary pattern, participants were also asked to consume moderate amounts of fish and seafood, poultry, fermented dairy products and eggs whilst limiting their intake of processed foods, butter, and red and/or processed meats. Target serving sizes or quantities for the aforementioned food groups were provided during the baseline consultation, following randomization [42].

#### 2.4.3. Consultations and Resources

Participants received individualised education on their allocated dietary allocation during a 90 min baseline consultation, which included an opportunity to ask questions and discuss dietary recommendations as needed. Follow-up consultations were conducted fortnightly at weeks 2, 4, 6, 8, and 10 (30 min each), with a final session at week 12 lasting 60 min. The baseline and week 12 consultations were delivered in person, whereas follow-up consultations (weeks 2–10) were offered either in person or via Zoom, depending on participant preference. To ensure consistency in intervention delivery, when additional study sites were introduced (University of South Australia or Monash University), the baseline and week 12 consultations were provided remotely by the same Accredited Practising Dietitian via Zoom.

Each dietary consultation included structured dietary education, individualised dietary counselling, and goal setting principles using the SMART (Specific, Measurable, Achievable, Relevant, Time-bound) framework. Participants rated the importance of their goals, their confidence in achieving them, and provided self-assessed feedback. Tailored meal and recipe suggestions were also provided.

Consultations were supplemented with a suite of printed study resources, including health education materials, informational pamphlets, meal suggestions, recipes, a fridge magnet summarizing key dietary principles and shopping lists. An infographic outlining recommended food groups and portion sizes for each dietary protocol was provided (Supplementary Materials). Additionally, participants were given food checklists for self-monitoring, which were reviewed during consultations to guide dietary counselling and goal setting.

#### 2.4.4. Digital Messaging

Participants received weekly health prompts via text messages on their mobile phones. These one-way messages reinforced essential aspects of the intervention and provided practical strategies to support dietary adherence, such as “*Did you know legumes stabilise blood sugar levels while keeping us full? Need inspiration? Beans on toast, add lentils to a salad or snack on roasted chickpeas.” and “Having support from someone close to you can help you reach your goals. Spend time with someone who will encourage you to maintain a Mediterranean eating style*.”

### 2.5. Outcomes

#### Baseline Characteristics

Participant characteristics (e.g., age, education, household income, country of birth, smoking status, other medical conditions, use of supplements or medications) were collected at baseline. Additional data was collected throughout the study with outcome measures and timepoints presented in Table 2.

### 2.6. Feasibility

Guided by Pfledderer et al. [41], feasibility was assessed narratively, considering key factors related to recruitment, retention, and intervention implementation. An overview of the feasibility outcomes is presented in Table 3.

Participant acceptability and factors influencing dietary adherence at baseline and week 12 were assessed with the use of surveys and semi-structured interviews for participants randomised to receive the MedDiet intervention.

The development of the survey tools and the interview schedule were informed by the COM-B model and reviewed by the stakeholder advisory committee. Participant acceptability and lived experience were conducted solely within the intervention group due to the novelty of the dietary approach and the lack of existing research on adherence challenges for this approach in women with PCOS. As this data has been reported elsewhere [36], a brief description of the methods and a summary of the results will be included in the present feasibility study.

#### 2.6.1. Surveys

At baseline and week 12, participants completed a written survey assessing their confidence and perceived ability to overcome common barriers to dietary adherence, such as food access, nutrition knowledge, cooking skills, and motivation. The survey included eight statements rated on a 5-point Likert scale, with 1 representing “very low” or “no confidence/ability,” and 5 indicating “very high” confidence/ability. At week 12, participants also completed a survey evaluating the intervention’s delivery, including the usefulness and accessibility of the educational sessions, resources, and text messages, with responses rated from “strongly agree” to “strongly disagree.”

#### 2.6.2. Interviews

At week 12, participants participated in semi-structured interviews, conducted either in person or via Zoom, with the study Accredited Practising Dietitian (NS), who had experience in qualitative research. The interviews, lasting approximately 25 min, explored barriers and facilitators to following the MedDiet, using a question guide based on the COM-B model to assess capability, opportunity, and motivation. Memoing was conducted immediately after each interview to capture key contextual insights and enrich data interpretation.

#### 2.6.3. Dietary Intake

Dietary intake of all participants was collected using a four-day food record completed at baseline, week 6, and week 12. Participants were instructed to record all food and beverage intake over three weekdays and one weekend day, using standard household measures (e.g., cups, tablespoons, teaspoons) for portion estimation. The study Accredited Practising Dietitian cross-checked all records for completeness, and any ambiguities were clarified with participants. This data was used to assess the appropriateness of collecting dietary intake data in this study; however, in a larger, adequately powered study, dietary intake data would also be used as a secondary outcome measure to assess dietary compliance and undertake a comprehensive nutrient analysis using FoodWorks Professional (Xyris software, version 10. Brisbane, QLD, Australia) incorporating the AFCD 2019 and AUSNUT 2011–2013 databases.

#### 2.6.4. Mediterranean Diet Adherence

Adherence to the MedDiet was assessed at baseline and week 12 using the validated 14-item MEDAS questionnaire [44]. This tool evaluates adherence based on the frequency of consumption of 12 key dietary components and two food habits consistent with a traditional MedDiet. Each item in the questionnaire was dichotomously scored as either 0 or 1, producing a maximum score of 14. A score ≥10 was suggestive of high adherence, scores between 6 and 9 indicated moderate adherence, whereas a score ≤5 was considered low adherence.

### 2.7. Preliminary Measures

#### 2.7.1. Biochemical Measures

Preliminary analysis on the effectiveness of the MedDiet intervention was assessed using metabolic, hormonal and anthropometric parameters. Specifically, fasting venous blood samples were collected at baseline and week 12 at pathology collection centres. The pathology companies varied according to the study site and included, Queensland Medical Laboratory Pathology, Melbourne Pathology and Clinpath Pathology. Biochemical analyses included total testosterone, sex hormone-binding globulin, fasting glucose, and fasting insulin. Insulin sensitivity was estimated using the homeostatic model assessment (HOMA-IR) according to the following equation:

Insulin sensitivity = [fasting insulin (mU/L × fasting glucose (mmol/L)]/22.5 [48].

#### 2.7.2. Anthropometric Measures

Anthropometric and body composition measurements were collected at baseline and week 12. Body weight was measured to the nearest 0.1 kg without footwear or heavy clothing. Height was measured barefoot using a wall-mounted stadiometer to the nearest 0.1 cm. BMI was calculated as weight (kg) divided by height squared (m^2^). Waist circumference was measured to the nearest 0.1 cm using a flexible steel tape measure (Lufkin Executive Thinline) at the point midway between the iliac crest and the lower costal border (lower rib) according to standardized protocols [49]. This was measured in triplicate by trained research personnel, with the mean of the three measures used for analysis.

#### 2.7.3. Physical Activity

Participants were instructed to maintain their usual physical activity patterns throughout the study. To assess physical activity, at the baseline and week 12 assessments, participants completed the short-form International Physical Activity Questionnaire (IPAQ-SF) [50] which evaluates activity levels over the previous seven days.

### 2.8. Analysis

Participants who completed the baseline and week 12 follow-up assessments were included in the analysis, as per CONSORT recommendations [40]. Feasibility outcomes were reported narratively. Continuous variables were presented as means ± standard deviation (SD) or median and interquartile ranges (IQRs), and categorical data were presented as frequencies and percentages. Independent samples *t*-tests, chi-square analyses, or Fisher’s exact tests used to assess between-group differences. Paired *t*-tests were used to assess within-group changes over time and the Wilcoxon signed-ranks test was used to identify changes in survey responses. Quantitative analyses were conducted using SPSS for Windows 27.0 (IBM Corp., Armonk, NY, USA), with statistical significance set at *p* ≤ 0.05.

Qualitative data from audio-recorded interviews were transcribed verbatim and analysed using framework analysis in NVIVO software (QSR International, Melbourne, Australia, version 12), as previously described [42]. Open coding was used to identify and group codes into related categories, which were then applied to the full dataset and organized in a framework matrix to facilitate theme generation. Memos were used throughout to guide analysis. The analysis was led by one researcher (NS) with consensus checks by AV, who independently analysed 30% of the data.

### 2.9. Protocol Adjustments

Due to slower than anticipated participant recruitment (discussed below), ethical approval was obtained to extend the study’s initial end date from December 2022 to May 2024, allowing additional time for recruitment. At the same time, two additional study sites, Adelaide and Melbourne, were introduced in response to feedback that many interested individuals were unable to attend in-person appointments at the Sunshine Coast site. These cities were selected based on the availability of staff to assist with data collection, and having larger populations (compared with the Sunshine Coast site), and as such were expected to yield more eligible participants. These changes were implemented in the middle of participant recruitment. Additionally, after recruitment concluded, the study’s primary aim was revised to focus on the feasibility, acceptability and adherence to a MedDiet intervention, with preliminary assessments of hormonal, metabolic, and anthropometric outcomes repositioned as secondary outcomes. This adjustment was made in response to lower than anticipated participant recruitment, which meant the study was underpowered and unable to detect meaningful changes in biochemical outcomes, in particular HOMA-IR. All modifications were agreed upon by the research team.

## 3. Results

### 3.1. Study Characteristics

**Figure 1:**
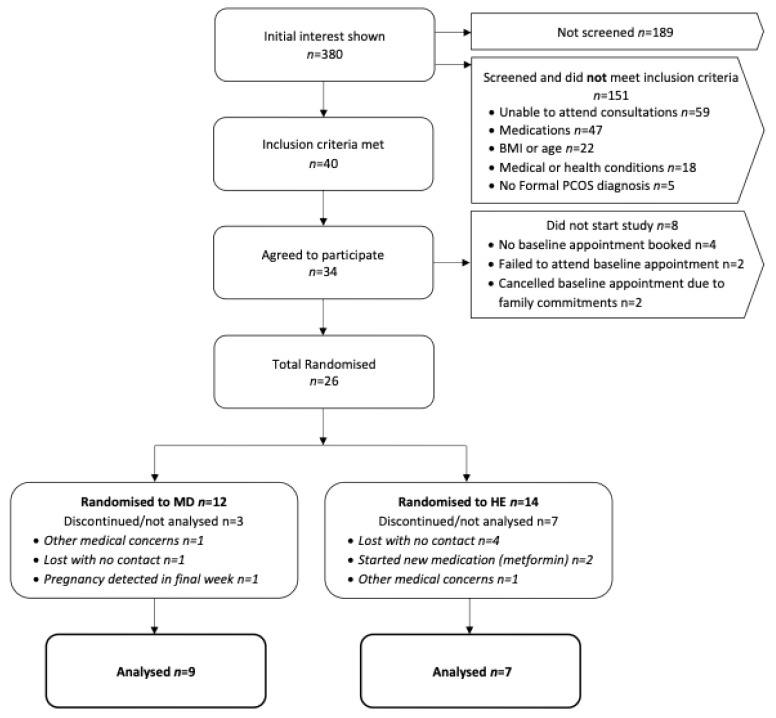
Flow chart of participant recruitment and reasons for exclusion. **Abbreviations:** BMI, body mass index; MD, Mediterranean diet; HE, Healthy Eating.

A total of 26 participants (29.0 ± 4.4 years; BMI: 38.8 ± 7.8 kg/m^2^) were enrolled in the study (*n* = 12 MedDiet; *n* = 14 HE), with the participant flow outlined in Figure 1. Baseline characteristics are summarised in Table 4. No significant differences in baseline characteristics between the two groups were identified.

A minority of participants (*n* = 5, 19%) reported a regular use of prescribed medications related to specific health conditions including: depression (*n* = 2), epilepsy (*n* = 1), reflux (*n* = 2), and asthma (*n* = 1). Almost half (*n* = 12, 46%), reported supplement use, including vitamins, minerals, and herbal formulations. Common supplements included vitamins C, D, and E, calcium, iron, magnesium, turmeric, inositol, collagen, NAC, CoQ10, fish oil, probiotics, and liver detox formulations. Most participants (85%) were Australian born, and 77% had a household income below AUD 150,000. Nearly half (46%) held a university qualification.

### 3.2. Feasibility

A summary of results for each aspect of the feasibility outcomes are presented in Table 5.

### 3.3. Findings Related to Recruitment

A total of *n* = 26 participants were enrolled in the study (MedDiet: *n* = 12; HE: *n* = 14) which was 60% of the intended sample size target (*n* = 42) (Figure 1). Over a 30-month period, a total of *n* = 380 women initially expressed interest in the study; however, half (*n* = 189, 50%) did not proceed to formal screening. The reason for not proceeding is unknown as these women did not respond to the researcher’s attempts to communicate. However, as each interested individual received a detailed study summary from the research team, it is possible women were able to self-exclude based on the study information (e.g., eligibility criteria, dietary protocol, etc.) that was provided. Among those who engaged in the screening process, *n* = 59 (31%) interested individuals were unable to attend in-person assessments due to geographical constraints. Recruitment was expanded to include two additional city locations to mitigate this issue, resulting in two participants enrolling at the Melbourne site (Monash University) and three at the Adelaide site (University of South Australia). Notably, two participants travelled significant distances to take part in the study, including 900 km and 400 km, respectively. A total of *n* = 40 women (11% of total interested individuals) met the inclusion criteria; however, *n* = 14 (35%) declined to participate before randomisation stating personal commitments (*n* = 4, 29%), international travel (*n* = 1, 7%), awaiting medical clearance (*n* = 1, 7%) and no response to research communication (*n* = 8, 57%). Of the *n* = 34 (85%) that initially agreed to participate, *n* = 4 (12%) did not respond to researcher communication before booking the initial baseline assessment, *n* = 2 (6%) scheduled appointments but failed to attend, and *n* = 2 (6%) booked appointments but later cancelled due to family commitments. This resulted in a total of *n* = 26 participants (65% of eligible individuals, 7% of total interested individuals) successfully enrolled in the study.

A variety of strategies were employed in the recruitment of potential participants. The majority of interest (87%) came from PCOS-focused Facebook support groups. A smaller proportion (6%) of participants were recruited from a database of individuals who had previously participated in PCOS related research and paid social media advertisements (2%). Other strategies included internal emails at the University of the Sunshine Coast (2%), paid advertising at health expos (1%), community notice boards (1%), community presentations (0.5%), and promotion through a women’s GP clinic (0.5%).

### 3.4. Findings Related to Inclusion/Exclusion Criteria

#### 3.4.1. Medications

During screening, many interested individuals (*n* = 47) were taking one or more medications that fell under the study’s exclusion criteria. These included metformin (*n* = 25), hormonal contraceptives (*n* = 19), glucagon-like peptide −1 receptor agonists (*n* = 5), and in vitro fertilisation (IVF) medications (*n* = 3). The exclusion of these medications was necessary due to their direct impact on the study’s biochemical outcomes.

#### 3.4.2. BMI

The eligibility criterion required participants to have a BMI of ≥25 kg/m^2^. There were *n* = 18 individuals who were excluded from participation as they did not meet this criterion (e.g., presented with BMI < 25 kg/m^2^)

#### 3.4.3. Age

Few women who expressed interest (*n* = 4) were greater than the age range criterion of 18–45 years, reflective of reproductive-age women.

#### 3.4.4. Medical Conditions

Several interested individuals had medical conditions that fell outside the study’s eligibility criteria, including type 2 diabetes (*n* = 4), Hashimoto’s disease (*n* = 3), thyroid disorders (*n* = 3), active cancer (*n* = 1), and type 1 diabetes (*n* = 1). These conditions were excluded due to their potential impact on the biochemical outcomes of interest for the present study. Additionally, four individuals who had recently undergone bariatric surgery and were actively losing weight were also excluded from participation. Two individuals self-excluded prior to randomisation due to a history of eating disorders. Lastly, four individuals were unable to obtain confirmation of a PCOS diagnosis and were therefore ineligible for the study.

### 3.5. Findings Related to Retention, Attrition and Adherence Rates to Study Procedures

Of the *n* = 26 randomised participants, *n* = 10 (38%) participants (MedDiet: *n* = 3; HE: *n* = 7) withdrew from the study or were not included in the final analyses (Figure 1). In the HE group (control), *n* = 7 (50%) participants completed the study, while the remaining withdrew. There were no significant differences in attrition rates between study groups (*p* = 0.11). Overall attrition was relatively consistent with the expected 30%, with a higher attrition rate observed in the control group (50%) compared to the intervention group (25%). Among those who did not complete the intervention, the majority (*n* = 4, 57%) withdrew without contact, *n* = 2 (29%) became ineligible after commencing metformin (an established exclusion criterion), and *n* = 1 (14%) withdrew due to other medical reasons. In the MedDiet group (intervention), *n* = 9 (75%) participants completed the entirety of the study. Of the *n* = 3 participants whose data were not included in the final analysis, *n* = 2 (67%) withdrew either without contact (*n* = 1; 33%) or due to other medical reasons (*n* = 1; 33%). The third participant completed the study but reported a positive pregnancy test at week 12, resulting in the exclusion of the participants metabolic, hormonal and anthropometric data. Additionally, two participants (one from each group) initially withdrew but later expressed interest in rejoining the study. However, both were lost to contact for a second time, before scheduling appointments.

In relation to attendance at the fortnightly dietary consultations, eight participants in the MedDiet group attended all sessions, while one participant missed two consultations at weeks 4 and 6, resulting in a 96% compliance rate. In the HE group, all participants who completed the study attended every scheduled consultation, achieving 100% compliance. Strategies implemented by the researcher to facilitate attendance and engagement included, appointment reminder emails and text messages, flexible booking/rescheduling options and building rapport through continuity of staff. Both in-person and online consultation options were provided, with all participants opting for the online option. Additionally, clinic notes from all consultations show participants demonstrated a willingness to implement dietary changes by actively setting and progressing their personal nutrition related goals.

### 3.6. Findings Related to MedDiet Adherence

**Figure 2:**
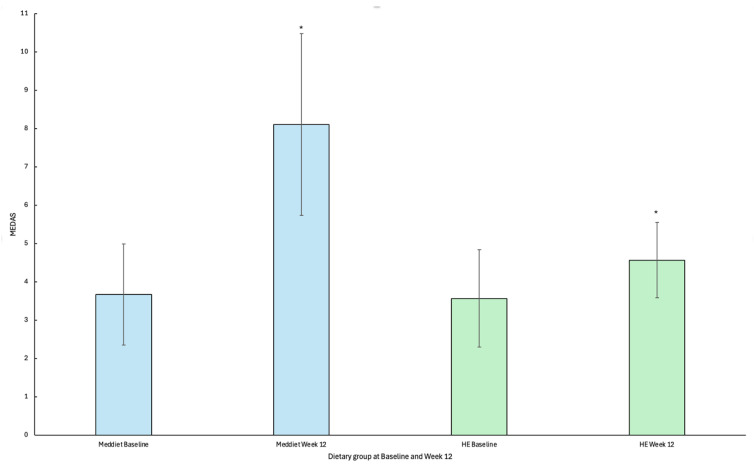
Mean change and standard deviation in Mediterranean diet adherence screener (MEDAS) scores by dietary group from baseline to week 12. * Statistical significance *p* < 0.05. **Abbreviations:** MedDiet; Mediterranean diet, HE; Healthy Eating, MEDAS; Mediterranean diet adherence screener.

Adherence was significantly greater in the MedDiet group than the HE group at week 12 (8.1 ± 2.4 vs. 4.6 ± 1.0; *p* = 0.002). Adherence to a MedDiet increased significantly from baseline to week 12 for participants allocated to receive the MedDiet intervention (*n* = 9) (3.67 ± 1.32; 8.11 ± 2.37; *p* ≤ 0.001; Figure 2). Participants randomised to the HE intervention showed a small, yet significant increase in MEDAS score from baseline to week 12 (*n* = 7) (3.57 ± 1.27; 4.57 ± 0.98; *p* = 0.02 (Figure 2).

### 3.7. Findings Related to the Suitability of Data Collection Procedures and Measures

The baseline and week 12 study visits were the only mandatory in-person appointments. These 90 min appointments were conducted at all University clinic sites, with one hour required for data collection and 30 min for dietary counselling. Arranging appointment times were challenging for some participants as the majority worked full-time or had caring responsibilities (*n* = 23, 88%). Therefore, appointments had to be scheduled across weekends, early mornings or later in the afternoon to account for competing commitments. The availability of appointments varied across study sites. The University of the Sunshine Coast site was the most flexible, offering appointments both during and outside of usual business hours, including weekends. In contrast, the Melbourne and Adelaide sites faced limitations due to staffing and clinic availability. The in-person component for participants at the Melbourne and Adelaide clinic sites was reduced to 15 min which allowed the trained Accredited Practising Dietitian to perform anthropometric measures only. The remaining components of the data collection were subsequently conducted online via Zoom with the study Accredited Practising Dietitian, which also provided consistency of data collection throughout the study.

All participants consented to the collection of anthropometric and biochemical data. Questionnaires and surveys that were provided during assessments and consultations had a 100% completion rate. In contrast, however, 15 food records (31%) across 3 different timepoints (baseline, week 6 and week 12) were not returned, and were missing from data collection.

#### Preliminary Measures

Preliminary effects of the intervention on hormonal, metabolic and anthropometric outcomes are presented in Table 6. Upon study completion, no between -group differences were observed for any hormonal, metabolic or anthropometric parameter. However, a significant reduction in waist circumference was observed for participants randomized to receive the MedDiet intervention (mean change: 3.03 ± 3.46; *p* = 0.03).

### 3.8. Adverse Events

No adverse events were reported in either the intervention or control groups throughout the duration of the study.

### 3.9. Participant Acceptability

A detailed analysis of participant acceptability and lived experience for participants randomised to the MedDiet intervention has been published previously [36]. Here, we provide a brief summary of the key findings relevant to feasibility. Participants reported significant increases in their knowledge toward a MedDiet, confidence in preparing and cooking foods, and confidence in having the time needed to prepare meals. Their ability to afford and access recommended foods also improved. However, there were no significant changes in their intention to follow a MedDiet long-term, perceived ability to adhere, or social acceptability of the diet among friends and family.

Participants strongly endorsed the dietary consultations and study resources. All participants found the consultations useful and rated the study materials as easy to read, easy to understand, and helpful. Most agreed that the study resources supported dietary adherence. Weekly text messages were well-received, with most participants finding them helpful, although preferences for the number of messages varied. Half of the participants wanted more frequent messaging, whereas the remaining participants either did not require more messaging or remained neutral.

Thematic analysis of barriers and facilitators of the MedDiet intervention identified 19 themes mapped to the COM-B framework. Key facilitators included education and resources, simplicity of guidelines, organisation and planning, self-discipline, improved self-efficacy, improved relationship with food, willingness to adapt, continue to adhere, perceived health benefits, improved health, reduced weight pressure and an enjoyable experience. Key barriers included habitual diet and stress. Lastly, organisation/planning, culinary skills, external influences, time, cost and availability were identified as both barriers and facilitators, depending on the participant’s experience.

## 4. Discussion

This study assessed the feasibility of a MedDiet intervention using a pilot clinical trial on metabolic, hormonal and anthropometric outcomes in women with PCOS and a BMI ≥ 25 kg/m^2^. Findings from this pilot study identify challenges in participant recruitment, including eligibility criteria and an inability to attend face-to-face appointments and assessments. Overall attrition was 38% (MedDiet: 25%; HE: 50%), aligning with previous dietary intervention studies in PCOS [34] but higher than the anticipated 30%, particularly in the control group. The majority of all data collection methods were identified as suitable and acceptable; however, strategies are needed to improve completion and return of 4-day food records for assessment of dietary compliance and nutritional status. Our preliminary results showed a MedDiet intervention was not more efficacious when compared against a standard Healthy Eating control for reductions in key metabolic, hormonal and anthropometric parameters in PCOS; however, our study was underpowered. Additionally, participants demonstrated the ability to adhere to a MedDiet and reported high acceptability of the dietary intervention. As such, a larger RCT is feasible, albeit with appropriate modifications to overcome challenges related to recruitment, attrition and completion of outcomes related to food records. To our knowledge, this is the first study to explore the feasibility of a MedDiet intervention for women with PCOS and one of very few to explore the feasibility of any dietary intervention in this population [51].

The strong response to study promotion indicates that a MedDiet intervention may be of interest to women with PCOS. However, a significant burden was placed on researchers to communicate and screen with the large number of interested individuals. This could be minimised in future studies through strategies including digital self-screening tools. Despite widespread interest, only 26 participants were enrolled, limiting the sample size and clinical utility of findings related to metabolic, hormonal and anthropometric parameters. Challenges in attending face-to-face appointments/assessments were also noted. This is likely attributed to Australia’s vast size and long travel times, though some individuals travelled significant distances (400 km and 900 km), demonstrating this barrier can be overcome. Additionally, competing commitments, including work and family, may have also limited participation. These factors align with previous research indicating time constraints, lack of social support, and work priorities affect participation and attrition in lifestyle interventions [52].

Since the recruitment target of 42 participants was not achieved (60%; *n* = 26), we suggest the study’s eligibility criteria may have been too restrictive. Specifically, only 40 participants (11% of those interested) were assessed as eligible. One key exclusion criterion was use of hormonal and insulin-sensitising medications, including metformin, which are commonly used as pharmacological agents in management of PCOS symptoms [1]. Future studies could consider broadening inclusion criteria and include these participants, with statistical adjustments considered to account for its effects. Given the common use of these medications in PCOS, their inclusion would better reflect the heterogeneity of the population. In the present study, the most successful recruitment strategy was PCOS support groups on social media. This generated the highest engagement, supporting evidence that targeted online communities effectively reach individuals with lived experience of the condition. This aligns with systematic reviews and meta-analyses indicating that recruitment strategies increasing participants’ awareness of a health condition and its potential impact improve recruitment rates [53]. Future strategies could include partnerships with PCOS organisations.

High attrition is frequently reported in dietary intervention studies involving women with PCOS [54]. In the present study, attrition was higher than anticipated (38%), albeit consistent with previous dietary interventions [39,55]. High attrition is typically attributed to mental, physical, and practical barriers, including time constraints and family responsibilities [54,56]. In our study, attrition was higher (50%) in the control compared to the MedDiet group (25%) (albeit not of statistically significant). While the reason for this is unclear, it may suggest a strong interest in the MedDiet as a novel dietary approach for PCOS management. This aligns with previous findings suggesting that women with PCOS prefer dietary interventions they feel are tailored to manage their health concerns [19,20]. Although participants in the HE group received the same level of support, including one-on-one individualized education, resources and counselling, it is possible that the MedDiet intervention was the primary motivator for attracting initial interest and a subsequent randomization to the HE group blunted interest and motivation of participants, contributing to greater attrition. Future retention strategies should consider modifications to the intervention delivery and recruitment, such as digital apps for content delivery, self-monitoring and data collection, flexible delivery formats and digital recruitment strategies [57].

Adherence and engagement with the intervention were evident, as participants maintained high attendance rates, demonstrating a willingness to engage in dietary change. This may be attributed to the flexibility of offering online consultations as previous research identified inconvenient session times and difficulty following session structure as potential barriers to engagement [58]. Dietary adherence was also positive as demonstrated by significant increases in MEDAS scores (increasing from low to moderate adherence) among participants randomized to receive the MedDiet intervention. This indicates participants were able to successfully adopt and adhere to key principles of a MedDiet. Although no significant between-group differences were observed for any hormonal, metabolic or anthropometric parameters, we did show significant reductions in waist circumference (mean change: 3.03 ± 3.46; *p* = 0.03) amongst participants randomized to the MedDiet intervention. However, no reductions were observed for any cardiometabolic parameter or other anthropometric measure for those randomized to the MedDiet intervention. There is meta-analytical evidence to show that MedDiet interventions (independent of caloric restriction), when combined with exercise, improve body composition outcomes, including reductions in waist circumference [30,59]. Nevertheless, this finding remains less clear when examining the independent effects of the MedDiet without exercise [59]. Although participants were instructed to maintain their usual physical activity behaviours throughout the intervention, it is possible participants randomized to the MedDiet increased their levels of physical activity throughout the intervention. Moreover, reductions in waist circumference, outside of caloric restriction, could be related to a number of key features related to the composition of the MedDiet including higher post-prandial fat oxidation rates due to the higher intake of monounsaturated fatty acids (MUFAs) [60], preferential oxidation of favourable fatty acids, such as oleic acid (found in olive oil) [61], or the high antioxidant and anti-inflammatory properties of the MedDiet supporting a more favourable metabolic milieu [62]. Nevertheless, given that no concurrent reduction in any other anthropometric or cardiometabolic parameters were observed, it is also possible that any observed reduction in waist circumference observed in the present study was attributable to measurement variability.

Of note, the HE group also showed significant increases toward adherence to a MedDiet, which is likely attributable to an improvement in overall diet quality. This finding is not unexpected given the overlap between key food group recommendations across both dietary interventions, including fruit, vegetables and wholegrain cereals. Nevertheless, both interventions remain distinct with the MedDiet strongly encouraging the consumption of key staples, including EVOO, legumes and nuts which were emphasized less in the HE group.

Data collection was feasible for all measures related to metabolic, hormonal and anthropometric parameters which required face-to-face appointments with a trained Accredited Practising Dietitian or phlebotomist (e.g., for blood taking). The impact of these appointments on retention was difficult to assess, as many participants who dropped out of the study did not respond to follow-up communications. However, it is possible the face-to-face visits for baseline and week 12 assessments may have contributed to attrition [53]. Self-reported anthropometric measures are a possible alternative; however, accuracy is questionable, particularly for measures such as waist circumference [63,64]. Future studies could provide visual instructions to improve accuracy [63,64] which may also reduce research staff burden and expand recruitment to a wider geographical area. Participant burden was also a likely factor when collecting dietary intake data using 4-day food records across multiple study timepoints, with a 31% rate of missing data. Although participant burden could be eased by reducing number and frequency of recording days (e.g., 3-days at two study timepoints), digital food tracking apps, such as Easy Diet Diary (Xyris Software, Brisbane, QLD, Australia), may also provide a more convenient alternative while still maintaining research rigor [21] where analysis can be performed using professional software (e.g., FoodWorks10^®^) [65], reducing participant and researcher burden. Alternatively, automated 24 h recalls can also be completed and analysed through web-based applications, also providing a convenient method for dietary collection and analysis [66].

Participant acceptability and lived experience were explored among those randomised to the MedDiet intervention only [36]. Participants reported they enjoyed following a Mediterranean-style diet, with the intervention increasing their confidence in their dietary knowledge and ability to carry out practical aspects such as cooking and meal preparation. Participants also reported the personalised consultations, and tailored resources were easy to understand which may have also facilitated adherence. Although barriers to dietary adherence were identified, including time, cost, culinary skills, organisation/planning and stress, many were addressed throughout the consultations and in conjunction with the practical resources. One barrier, however, that was not addressed was stress. Incorporating techniques and strategies for stress management would be a valuable addition for future interventions. Discussing barriers and facilitators to lifestyle change is a recommended practice point in clinical guidelines, alongside behavioural support strategies such as goal setting, self-monitoring and problem solving [18]. These findings suggest that a MedDiet may represent a viable and sustainable dietary strategy for women with PCOS, particularly when intervention components are designed to overcome both practical and motivational barriers [36].

## 5. Limitations

A limitation of this study is that we did not pre-define benchmarks to assess study feasibility, choosing instead to explore study characteristics to inform future study design. However, employment of a feasibility framework ensured a comprehensive examination of the results to inform future study design [36]. Moreover, due to recruitment constraints of this pilot study, our study was underpowered and fell short of the planned sample size (*n* = 42). As such, the clinical utility of the study findings related to the effects of a MedDiet on hormonal, metabolic and anthropometric parameters still remain unclear. While positive results for acceptability and adherence to the study protocol are promising, the exploration of participant lived experience for the intervention, its delivery and study resources were performed exclusively in participants randomized to the MedDiet intervention, making it difficult to ascertain if the belief is unanimous among all participants. Furthermore, our study was not without attrition bias, with a higher proportion of dropouts among participants randomized to the HE group. Nevertheless, due to the importance of establishing sustainable and efficacious options for the dietary management of PCOS, an adequately powered study is still warranted.

## 6. Conclusions

A research priority of the international evidence-based guidelines for PCOS management is to identify best practice for the lifestyle management of PCOS. Therefore, our study provides valuable knowledge and preliminary evidence related to the acceptability and feasibility of a Mediterranean-style dietary intervention. Our findings suggest that a MedDiet intervention was safe and accepted amongst women with PCOS and a BMI ≥ 25 kg/m^2^ as evidenced by increased adherence scores, high consultation attendance, and favourable participant feedback. As such, nutrition professionals should feel encouraged about the potential role of the MedDiet as a therapeutic dietary intervention for PCOS management, provided appropriate behavioural strategies are integrated into dietary counselling to address individual barriers which may impede adherence. Whilst our work provides preliminary insight, much larger and adequately powered clinical trials are needed to inform clinical practice guidelines, which also consider feasibility issues identified in the present study, including participant recruitment, attrition and the collection of dietary intake data.

## Figures and Tables

**Table 2:** Overview of data collection.

Variable	Baseline	Week 6	Week 12
Age	x		
Education	x		
Household income	x		
Country of birth	x		
Smoking status	x		
Medical conditions	x		
Supplement use	x		
Medication use	x		
IPAQ-SF	x		x
4-day food record	x	x	x
MEDAS	x		x
TT	x		x
SHBG	x		x
FG	x		x
FI	x		x
HOMA-IR	x		x
Waist circumference	x		x
Weight	x		x
BMI	x		x

**Abbreviations**: MEDAS: Mediterranean diet adherence screener; TT: total testosterone; SHBG: sex hormone-binding globulin; FG: fasting glucose; FI: fasting insulin; HOMA-IR: homeostatic model assessment of insulin resistance; BMI: body mass index; IPAQ-SF: International Physical Activity Questionnaire—Short Form.

**Table 3:** Overview of feasibility outcomes.

Feasibility Outcome	Description
Recruitment metrics	Recruitment rate Eligibility rate
Retention and adherence	Completion rate Consultation attendance
Dietary adherence	Mediterranean diet adherence screener
Suitability of data collection	Completion of 4-day food records Consent to anthropometric measures Consent to biochemical measures Preliminary outcomes for anthropometric and biochemical measures
Safety	Reported adverse events
Acceptability	Participant acceptability and factors influencing dietary adherence were collected at baseline and week 12 through surveys and semi-structured interviews for participants randomised to receive the MedDiet intervention. This study has been published elsewhere [42]; however, a summary of study findings will be provided within the results of the present study. The methods for survey and questionnaire development are provided below.

**Table 4:** Baseline characteristics of participants by study group.

Characteristic	MedDiet (*n* = 12)	HE (*n* = 14)
Age (years)	30.0 ± 5.5	28.2 ± 3.1
MEDAS	4.4 ± 2.0	3.5 ± 1.0
Total testosterone (nmol/L)	1.5 ± 0.4	1.4 ± 0.5
SHBG (nmol/L)	28.9 ± 12.3	27.4 ± 11.4
Fasting insulin (mU/L)	15. 2 ± 4.7	19.1 ± 16.1
Fasting glucose (mmol/L)	5.0 ± 0.5	4.9 ± 0.3
HOMA-IR	3.4 ± 1.2	4.2 ± 4.0
Weight (kg)	101.7 ± 18.8	113.8 ± 23.0
BMI (kg/m^2^)	37.1 ± 7.0	40.3 ± 8.3
Waist circumference (cm)	106.0 ± 15.9	113.6 ± 15.4
Highest level of education		
Year 10	0 (0)	1 (7)
Year 12	1 (8)	4 (29)
Trade	3 (25)	5 (36)
Bachelor	8 (67)	4 (29)
Country of birth		
Australia	9 (75)	13 (93)
Brazil	1 (8)	0 (0)
India	1 (8)	0 (0)
Argentina	1 (8)	0 (0)
New Zealand	0 (0)	1 (7)
Household income (AUD)		
<75,000	3 (25)	2 (14)
75,000–150,000	7 (58)	8 (57)
>150,000	2 (16)	4 (28)
Smoking status		
Current smoker	1 (8)	0 (0)
Never smoked	8 (67)	11 (79)
Former smoker	3 (25)	3 (21)
Other health conditions		
Yes	6 (50)	9 (64)
No	6 (50)	5 (36)
Taking medications		
Yes	2 (17)	3 (21)
No	10 (83)	11 (79)
Taking supplements		
Yes	4 (33)	8 (57)
No	8 (67)	6 (43)

Data reported as mean ± SD or frequencies and percentages. **Abbreviations:** BMI, body mass index. MEDAS, Mediterranean diet adherence screener.

**Table 5:** Feasibility outcomes.

	Ad Libitum Mediterranean Diet or Healthy Diet Intervention	Recommendations for Full-Scale Clinical Trial
Recruitment metrics		
Eligibility rate	40 (11% of interested individuals)	Incorporate digital self-screening tool for interested participants to determine their eligibility before contacting research team. Record reasons for ineligibility or reason for disinterest. Consider removing need for an in-person appointment by including self-reported anthropometric data. Consider expanding inclusion criteria to include insulin sensitising and hormonal contraceptives.
Recruitment rate	26 participants enrolled over 30 months	Strategies to improve recruitment may include multiple study locations and flexible appointments, partnering with PCOS support groups and clinical settings to promote the study, and flexible inclusion criteria.
Retention and adherence		
Completion rate	16 (62%) completion 9 (75%) MedDiet 7 (50%) HE	Consider telephone check-ins on the weeks alternate to the consultations to provide more consistent contact, interactive digital messages to optimise engagement.
Consultation attendance	96% for MedDiet 100% for HE	Continue to offer telehealth options.
Dietary adherence		
MEDAS	Significantly increased throughout study (MedDiet; Baseline: 3.67 ± 1.32; Week 12: 8.11 ± 2.37; *p* ≤ 0.001; HE; Baseline: 3.57 ± 1.27; Week 12: 4.57 ± 0.98; *p* = 0.02)	
Suitability of Data Collection		
Completion of 4-day food records	69%	Consider offering alternatives such as a digital app for completing food record, 24 h automated recall, shorter and/or less frequent checklists.
Consent to anthropometric measures	100%	
Consent to biochemical testing	100%	
Preliminary outcome measures	Waist circumference reduced significantly over the course of the study for the MedDiet group Other measures were mixed and not significant	
Safety and tolerability		
Adverse events	No adverse events	
Participant acceptability of Mediterranean diet intervention		
Summary of acceptability and lived experience study [31]	Participants reported: Increases in confidence and ability to following a MedDiet; Consultations and resources made it easier to adhere; Barriers and facilitators to dietary adherence were identified.	Ensure consultations and participant resources continue to focus on practical strategies and increase motivation. Could incorporate more frequent digital messaging.

**Abbreviations**: MedDiet: Mediterranean diet; MEDAS: Mediterranean diet adherence screener.

**Table 6:** Biochemical and anthropometric mean change from baseline to week 12 by study group and *p* value for between-group differences.

	Mediterranean Diet				Healthy Eating				Between-Group Difference
Variable	Mean Change	SD	95% CI	*p*	Mean Change	SD	95% CI	*p*	*p*
TT	0.18	0.28	−0.03, 0.39	0.09	0.01	0.25	−0.21,0.24	0.88	0.24
SHBG	1.67	9.47	−5.62, 8.95	0.61	−0.29	8.30	−7.96, 7.39	0.93	0.67
FI	0.53	4.53	−2.95, 4.02	0.73	−1.71	6.80	−8.00, 4.57	0.53	0.44
FG	−0.78	0.32	−0.32, 0.17	0.49	−0.86	0.41	−0.46, 0.29	0.60	0.97
HOMA-IR	0.07	1.06	−0.35, −0.74	0.85	−0.43	1.55	−1.86, 1.00	0.49	0.46
Weight	1.92	4.44	−1.49, 5.33	0.23	0.60	3.10	−2.26, 3.46	0.63	0.51
BMI	0.68	1.63	−0.58, 1.93	0.25	0.20	1.18	−0.89, 1.29	0.67	0.53
WC	3.03	3.46	0.37, 5.70	0.03	0.41	3.38	−2.72, 3.54	0.76	0.15

Data reported as mean change ± SD. Significance *p* < 0.05. **Abbreviations**: TT: total testosterone; HOMA-IR: homeostatic model assessment-insulin resistant; FI: fasting insulin; FG: fasting glucose; BMI: body mass index; WC: waist circumference; SHBG: sex hormone-binding globulin.

## Data Availability

The data generated during and/or analysed in this study are available from the corresponding author upon reasonable request.

## Supplementary Materials

The following supporting information can be downloaded at: https://www.mdpi.com/article/10.3390/jcm14165842/s1. Table S1 Infographic outlining recommended food groups and portion sizes for each dietary protocol (MedDiet and HE).
